# *Afroprinus cavicola* gen. et sp. n. from the Afrotropical region with notes on cave-dwelling Saprininae (Coleoptera, Histeridae)

**DOI:** 10.3897/zookeys.294.4800

**Published:** 2013-04-22

**Authors:** Tomáš Lackner

**Affiliations:** 1 Czech University of Life Sciences, Faculty of Forestry and Wood Sciences, Department of Forest Protection and Entomology, Kamýcká1176, CZ-165 21 Praha 6 – Suchdol, Czech Republic

**Keywords:** Coleoptera, Histeridae, Saprininae, *Afroprinus*, Afrotropical, taxonomy

## Abstract

A new genus and species from Kenya, *Afroprinus cavicola* is herein described and illustrated and its systematic position is discussed. By the prosternal pre-apical foveae connected by marginal prosternal stria it resembles most of the Afrotropical species of the genus *Chalcionellus* Reichardt, 1932, or some species of the genus *Pholioxenus* Reichardt, 1932 from South Africa and Namibia. *Afroprinus* can be distinguished from *Chalcionellus* chiefly by the lack of pronotal depressions and a coarsely sculptured, non-metallic dorsum; from Afrotropical species of *Pholioxenus* it can be most easily distinguished by the asetose pronotal hypomeron. The new taxon was discovered in a cave, but lacks obvious troglophilic adaptations. Notes on other Saprininae taxa found in caves are given. An identification key to the genera of Afrotropical Saprininae is provided.

## Introduction

The Saprininae of the Afrotropical Region are quite well known and have been studied for many years, and except for descriptions of genera or species scattered in the entomological literature of the past 180 years there are several generic revisions (e.g. Gomy and Vienna 1996), as well as many studies on the histerid fauna of particular countries that also contain data on the Saprininae. Such country studies (in most cases called ‘Contributions to the knowledge’) were mostly published by Gomy (see e.g. Gomy 1978, 1983 or 1986), but also by other authors (e.g. Desbordes 1930 or Penati and Vienna 1996). Despite this, there is still no systematic revision of the Afrotropical Saprininae, or a catalogue to deal specifically with this region. According to the recent world catalogue of Mazur (2011) there are 22 genera and 199 species of Saprininae occurring in the Afrotropical region. However, it is probable that such a large tropical area must house much larger diversity of the Saprininae than the taxonomic literature indicates.

During a visit to the Natural History Museum, London, UK in 2009 I have examined a series of apparently unknown Saprininae specimens, identified as *Gnathoncus* sp. After having performed a phylogenetic analysis of the Saprininae subfamily (Lackner, unpublished), I can conclude that this is an unknown taxon belonging to a new genus. The prosternal pre-apical foveae connected by the marginal prosternal stria found in this taxon is a rare feature among the Old World Saprininae and it is more likely to be found among the members of *Euspilotus* Lewis, 1907, common to the Neotropical region (Lackner, pers. observ.). However, in the Afrotropical region there are Saprininae that have the prosternal pre-apical foveae connected by the marginal prosternal stria. These taxa are found among ill-defined and most likely polyphyletic genera *Chalcionellus* Reichardt, 1932 and *Pholioxenus* Reichardt, 1932. Revisions of both afore-mentioned genera are badly needed.

In this contribution to the systematics and taxonomy of the Saprininae a new genus and its type species are described and the systematic position of the new genus in the Saprininae subfamily is discussed. A tentative key to the genera of the Afrotropical Saprininae is provided.

## Material and methods

All dry-mounted specimens were relaxed in warm water for several hours or overnight, depending on the body size. After removal from original cards, the beetles were side-mounted on triangular points and observed under a Nikon 102 stereoscopic microscope with diffused light. Some structures were studied using methods described by Ôhara (1994): the head and male genitalia were macerated in a hot 10% KOH solution for about 15 minutes, cleared in 80% alcohol, macerated in lactic acid with fuchsine, incubated at 60ºC for two hours, and subsequently transferred into a mixture of glacial acetic acid 1 part and methyl salicylate 1 part heated at 60ºC for 15 minutes and cleared in xylene. Specimens were then observed in α-terpineol in a small glass dish. The mentum, labium, labrum, mandibles and antennae were disarticulated. Digital photographs of the male terminalia, mouthparts and antenna were taken by a Nikon 4500 Coolpix camera and edited in Adobe Photoshop CS4. Based on the photographs or direct observations, the genitalia, mouthparts and antennal structures were drawn using a light-box Hakuba klv-7000. SEM photographs were taken with a JSM 6301F microscope at the laboratory of Faculty of Agriculture, Hokkaido University, Sapporo, Japan. All available specimens were measured with an ocular micrometer. Morphological terminology follows that of Ôhara (1994) and Lackner (2010). Separate lines of the same label are demarcated by a slash (/). The following acronyms of museums and private collections are used throughout the text:

**NMH** Natural History Museum, London, UK (R. Booth);

**TLAN** Tomáš Lackner’s collection, temporarily housed at Naturalis Biodiversity Centre, Leiden, Netherlands.

**Abbreviations of body measurements (from Ôhara 1994) are as follows:**

**PEL** length between anterior angles of pronotum and apices of elytra

**APW** width between anterior angles of pronotum

**PPW** width between posterior angles of pronotum

**EL** length of elytron along sutural line

**EW** maximal width between outer margins of elytra.

## Results

### Afroprinus gen. n.

urn:lsid:zoobank.org:act:BD1A264E-6EDB-4F69-A2D7-5EB0FBB57583

http://species-id.net/wiki/Afroprinus

#### Type species:

*Afroprinus cavicola* sp. n.

#### Diagnosis.

Body length 2.125–2.375 mm, dorsal surface entirely punctate; cuticle dark brown to black, without metallic luster, frontal and supraorbital striae present, well developed; sensory structures of antennal club in form of small sensory area and corresponding vesicle situated on internal distal margin; pronotal hypomeron asetose; elytral disc with four long carinate dorsal elytral striae, fifth stria occasionally present on fourth elytral interval; apex of prosternal process convex, prosternal pre-apical foveae large and deep, connected apically by marginal prosternal stria; carinal prosternal striae shortened apically, not united anteriorly; lateral prosternal striae terminating in large deep prosternal pre-apical foveae; meso-metaventral sutural stria present, undulate. Venter asetose.

#### Differential diagnosis.

The general appearance of *Afroprinus* somewhat resembles that of *Hypocacculus*, differing chiefly in the large prosternal pre-apical foveae connected by the deep marginal prosternal stria and in the marginal pronotal stria that in dorsal view is visible along its entire length. By the convex apical third of the prosternal process and by the prosternal pre-apical foveae connected by the marginal prosternal stria *Afroprinus* can also be confused with several Afrotropical species of the genera *Chalcionellus* or *Pholioxenus*. It differs from those species of *Chalcionellus* that have the prosternal pre-apical foveae connected by the marginal prosternal stria by the sculpture of dorsal surface, which is metallic and much coarser in *Afroprinus* and by lacking the pronotal depressions of *Chalcionellus*. From those species of Afrotropical *Pholioxenus* (mainly from South Africa and Namibia) that likewise have their prosternal pre-apical foveae connected by marginal prosternal stria, *Afroprinus* differs by the asetose pronotal hypomeron and much coarser and denser elytral punctuation.

#### Biology.

The type series of *Afroprinus cavicola* has been found on bat droppings in a Kenyan cave.

#### Distribution.

Kenya.

#### Etymology.

The generic name is a combination of the genus name ‘*Saprinus*’ with a prefix derived from the continent of Africa. Gender masculine.

### Afroprinus cavicola sp. n.

urn:lsid:zoobank.org:act:BE636DF4-CDA8-40D8-98F2-8ECA078706FD

http://species-id.net/wiki/Afroprinus_cavicola

Figs 1 21

#### Type locality.

Kenya, Chyulu Hills.

#### Type specimens examined.

Holotype, ♂, side-mounted on a triangular mounting card with male genitalia glued to the same card; “KENYA: / Chyulu Hills / Univ. of Nairobi” [written]; “bat droppings / in cave” [written]; “Brit. Mus. / 1972-215” [printed-written]; “*Gnathoncus* sp. / P.M. Hammond / det. 1972” [written-printed]; “09-071” [yellow label, pencil-written, added during the present study]; “*Afroprinus* / *cavicola* / n. gen. & sp. / HOLOTYPE / det. T. Lackner 2010” [red label, written] (NMH). Paratypes, 4 ♂♂ and 2 ♀♀, idem, but without the identification label by P.M. Hammond (NMH; 1 ♂ PT in TLAN).

#### Description.

Male and female.Body length: PEL: 2.125–2.375 mm; APW: 0.75–0.875 mm; PPW: 1.625–1.75 mm; EL: 1.375–1.50 mm; EW: 1.875–2.00 mm.

**Figures 1–11.**
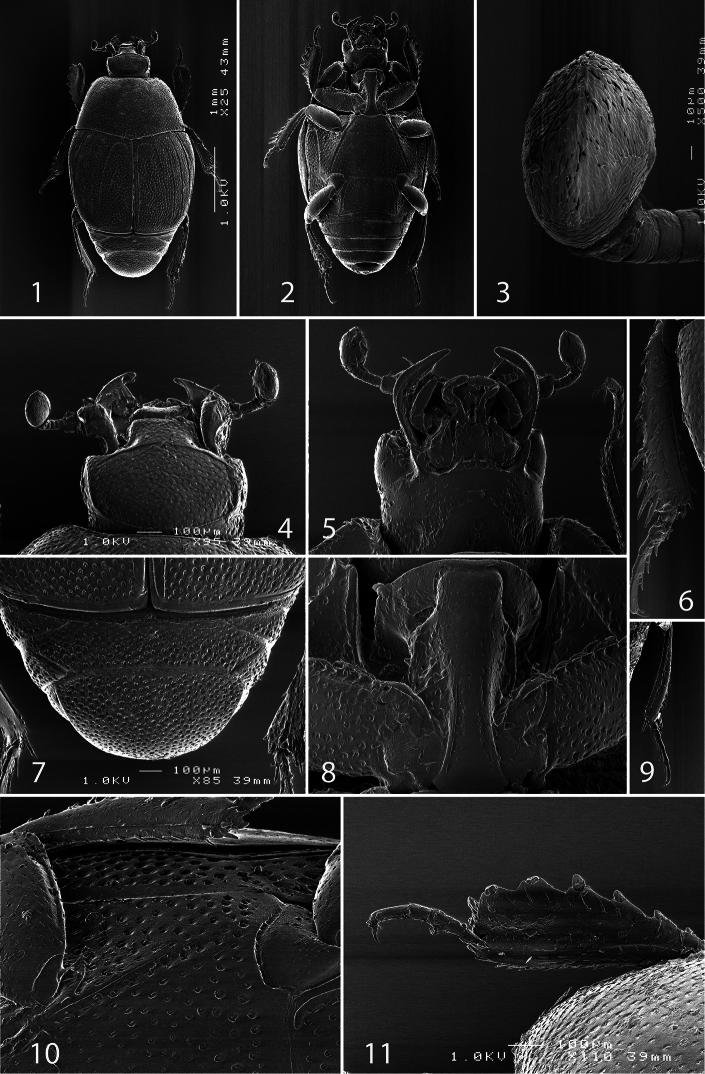
*Afroprinus cavicola* gen. et sp. n., SEM micrographs. **1** habitus, dorsal view **2** ditto, ventral view **3** antennal club, dorso-lateral view **4** head, dorsal view **5** ditto, ventral view 6 mesotibia, dorsal view **7** propygidium and pygidium **8** prosternum **9** metatibia, dorsal view **10** lateral disc of metaventrite + metepisternum **11** protibia, dorsal view.

Body (Figs 1, 2) ovoid, convex, ventral surface slightly flattened, cuticle matte, dark brown; legs, mouthparts and antennomeres I and II rufous; remaining part of antenna somewhat lighter.

Antennal scape (Fig. 4) with several short setae; antennal club (Figs 3, 14) round, without visible articulation, somewhat flattened dorso-ventrally; approximately distal half of its surface with thick short sensilla; proximal half with imbricate microsculpture, sensilla absent; sensory structures of antennal club (Fig. 14) with tiny sensory area accompanied by a tiny stipe-shaped vesicle on internal distal margin of antennal club.

**Figures 12–14.**
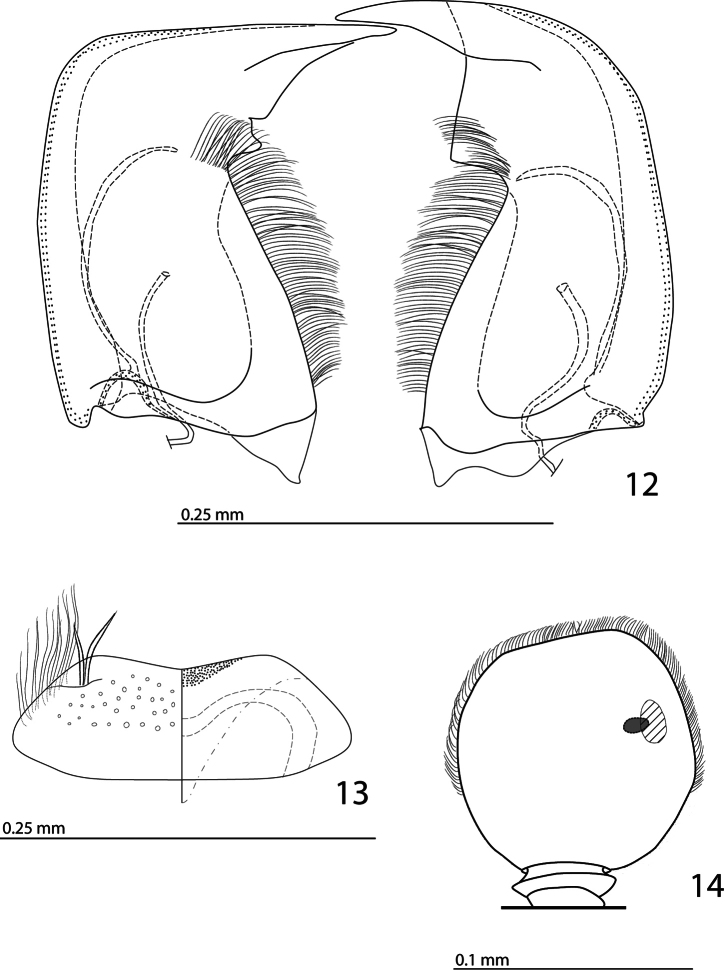
*Afroprinus cavicola* gen. et sp. n., mouthparts and sensory structures of the antenna. **12** mandibles, dorsal view **13** labrum, left half showing dorsal view; right half showing ventral view **14** antennal club showing sensory structures of the antenna

Mouthparts. Mandibles (Fig. 12) with rounded outer margin strongly curved inwardly, apically pointed; sub-apical tooth on inner margin of left mandible large, almost perpendicular; disc of labrum (Fig. 13) convex, labral pits each with two setae; terminal labial palpomere elongate, four times as long as broad; cardo of maxilla with several short setae, stipes triangular, with three long setae; mentum quadrate, anterior margin with deep median notch, anterior and lateral margins with a row of sparse short ramose setae, disc of mentum with several short setae; terminal maxillary palpomere (Fig. 5) elongate, five times as long as broad, approximately 2.5 times as long as penultimate palpomere.

Anterior margin of clypeus (Fig. 4) slightly elevated, surface punctate, slightly depressed medially; frontal stria well impressed, curved outwardly, carinate, complete; continued as carinate supraorbital stria; disc of frons (Fig. 4) entirely densely and coarsely punctate, punctures separated by spaces shorter than half of their diameter, sparser near margins; eyes flattened, visible in dorsal view.

Pronotal sides (Fig. 1) moderately narrowing anteriorly, anterior angles blunt; marginal pronotal stria complete, slightly carinate, visible along its entire length in dorsal view; pronotal disc entirely punctate, punctures separated by spaces 1-2 times as wide as puncture diameter; pronotal hypomeron setose; scutellum small, inconspicuous.

Elytra: epipleuron with fine scattered punctures; marginal epipleural stria complete; marginal stria straight, well impressed, carinate, continued as intermittent apical stria. Humeral stria well impressed on basal third, somewhat obliterated by coarse punctuation; inner subhumeral stria well developed, visible as long median fragment posteriorly nearly reaching first dorsal stria; with carinate dorsal striae 1-4 (some specimens with a vague fragment of fifth stria on fourth elytral interval); striae 1-3 sub-equal in length, posteriorly reaching approximately five-sixths of elytral length, fourth dorsal stria slightly shorter, anteriorly well-connected with carinate sutural stria; sutural stria straight, well impressed, posteriorly connected with fragmented apical stria; between sutural stria and suture with row of microscopic punctures. Entire surface coarsely and densely punctate, punctures separated by spaces sub-equal to their diameter or shorter, periscutellar area with slightly sparser punctuation; interspaces with isodiametric microsculpture.

Propygidium (Fig. 7) completely exposed, its punctuation similar to that on elytra and pygidium.

Antero-median margin of prosternum (Fig. 8) straight, rounded laterally; pre-apical foveae deep, connected by marginal prosternal stria; prosternal process apically convex, rounded; carinal prosternal striae (Fig. 8) almost parallel-sided, apically reaching approximately half-length of prosternal process; lateral prosternal striae carinate, terminating in large pre-apical foveae; entire prosternal process with scattered punctures.

Antero-median margin of mesoventrite straight; discal marginal mesoventral stria well impressed, emarginate anteriorly, complete; disc of mesoventrite with scattered round punctuation; meso-metaventral sutural stria undulate.

Intercoxal disc of metaventrite slightly convex, entirely covered with scattered fine punctures separated by spaces 2-3 times as wide as their diameter, anteriorly punctures becoming coarser and denser, in male more so; lateral metaventral stria (Fig. 10) straight, carinate, almost reaching metacoxa; lateral disc of metaventrite slightly impressed, with deep round punctures; metepisternum + fused metepimeron with distinctly denser punctures; marginal metepisternal stria complete, deeply impressed.

Intercoxal disc of first abdominal sternite with complete lateral striae, disc with scattered fine punctures, separated spaces as wide as 3 times puncture diameter.

Protibia (Fig. 11) slightly dilated; outer margin with 6 short teeth, each topped by short rounded denticle gradually reducing in size towards base of tibia, followed by a minute denticle; setae of outer row sparse, short; setae of median row even shorter; protarsal groove deep; anterior protibial stria carinate, shortened apically; protibial spur minuscule, approximate to tarsal insertion; outer part of posterior surface of protibia smooth, demarcated from median part by distinct straight line; posterior protibial stria complete, terminating in two inner posterior denticles, separating median part of posterior surface from smooth inner part of posterior surface; inner margin of protibia with dense row of lamellate setae that gradually increase in size towards tibial apex.

Mesotibia (Fig. 6) slender, outer margin with a single row of short denticles gradually increasing in size towards tibial apex; setae of outer row moderately long, sparse, lightly sclerotized; setae of median row much thinner and sparser; posterior stria almost complete and only slightly shortened distally; anterior surface with a row of short denticles on outer margin, surface otherwise smooth; anterior stria complete; apical spur short; apical margin of with several stout denticles; claws of terminal tarsomere slightly bent, shorter than half tarsomere length.

Metatibia (Fig. 9) more slender than mesotibia, its denticles sparser than those of mesotibia, otherwise similar to it.

**Figures 15–21.**
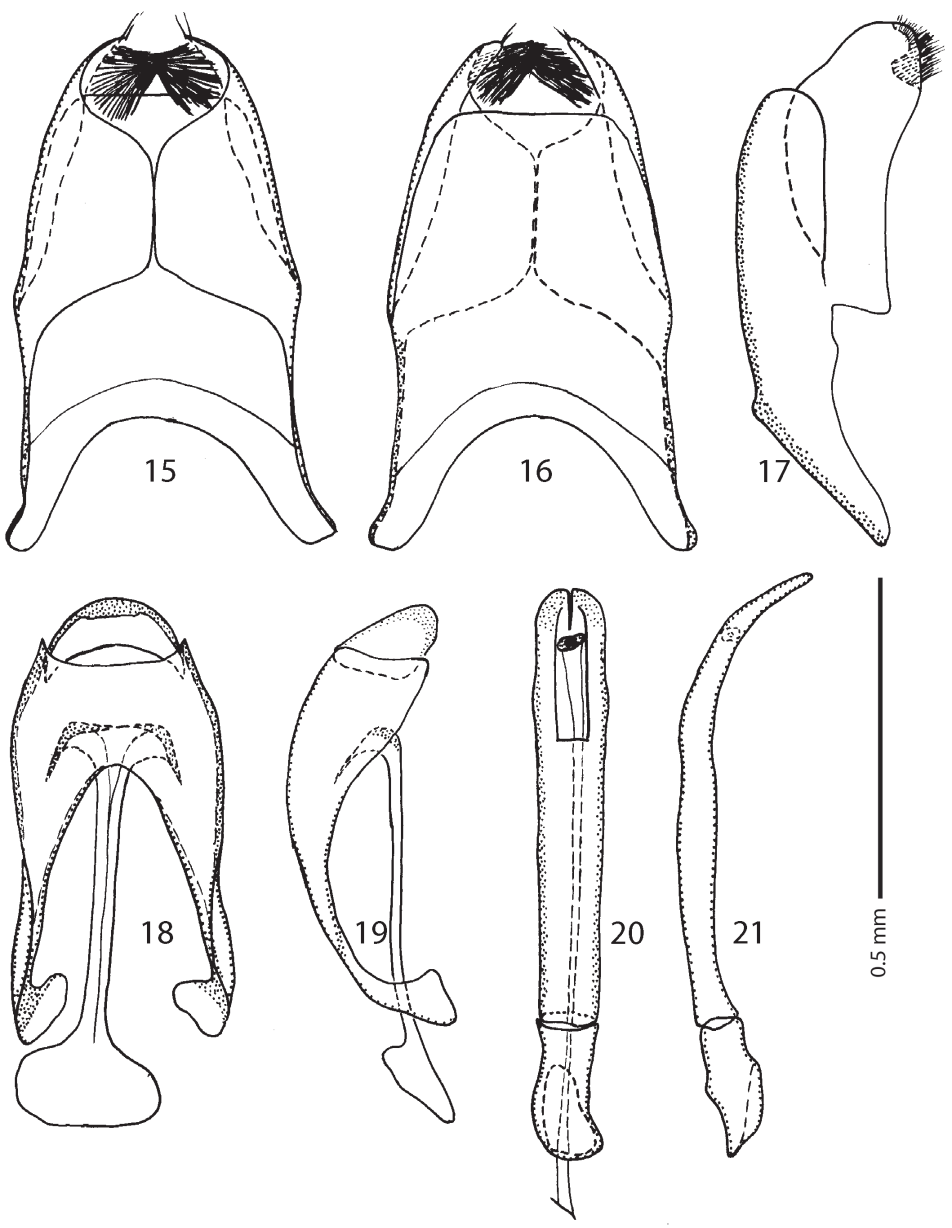
*Afroprinus cavicola* gen. et sp. n., male terminalia. **15** eighth sternite and tergite, ventral view **16** ditto, dorsal view **17** ditto, lateral view **18** ninth and tenth tergites, dorsal view; spiculum gastrale, ventral view **19** ninth, tenth tergites and spiculum gastrale, lateral view **20** aedeagus, dorsal view **21** ditto, lateral view.

Male genitalia: Eighth sternite (Figs 15–16) divided longitudinally; vela present, with dense brush of long setae; apex of eighth sternite with one or two short setae (Fig. 15); eighth tergite and eighth sternite fused laterally (Fig. 17). Ninth tergite (Fig. 18) not longitudinally divided medially; spiculum gastrale (Fig. 18) almost parallel-sided, abruptly dilated apically; basal end broadly rounded, spatulate. Aedeagus (Figs 20–21) almost parallel-sided, slightly broadening apically, in apical third curved ventrad; basal piece short, ratio of its length to length of parameres 1 : 4; parameres fused almost along their basal two-thirds.

#### Etymology.

The specific epithet was derived using a compounding method of word formation, by stringing together Latin word ‘caverna’ meaning cave and combining element of Latin origin ‘-cola’ (orig. *colo, ere* to inhabit) meaning inhabitant, referring to the cavernicolous habitat of this new species. ‘Cavicola’ is a noun in apposition, which is in accordance with ICZN rules; Article 11.9.1.2

##### Key to identification of Afrotropical genera of Saprininae

This key is preliminary and in future will be revised, especially in regard to the ill-defined and heterogeneous genera *Saprinus*, *Hypocacculus*, *Chalcionellus* and *Pholioxenus*. *Pholioxenus* shows a discontinuous distribution, with about two-thirds of its species living nidiculously inside burrows and faecal chambers of small ground mammals in the Palaearctic region and one-third found free-living in South Africa, Namibia etc., with a single species of unknown biology (*Pholioxenus trichoides* Kapler, 1992) described from north Sudan (Mazur 2011). Revision of the genus *Pholioxenus* is in preparation (Lackner, manuscript). Subgenera, with exceptions of *Hesperosaprinus* and *Neosaprinus* of the Nearctic and mainly Neotropical genus *Euspilotus* which are pertinent to this key, are excluded, since they fall within the limits of their respective genera. Limits of the Afrotropical region are according to Löbl and Smetana (2004) and encompass the entire continent of Africa south of Sahara, the island of Madagascar, along with Cape Verde Archipelago, São Tomé & Príncipe, Seychelles, Réunion, Comoros, island of Saint Helena and other smaller islands.

**Table:** 

1 (10)	Frontal and supraorbital striae completely absent (for fig. see Lackner 2010: fig. 302)^1^
2 (7)	Prosternal pre-apical foveae absent (for fig. see Lackner 2010: fig. 305)
3 (6)	Prosternum setose, elytral epipleuron setose, marginal elytral stria single
4 (5)	Lateral metaventral stria reaching metepisternum; all dorsal elytral striae 1-4 well developed; carinal prosternal striae strongly approximate, weak (absent in some specimens); lateral prosternal striae anteriorly joined by marginal prosternal stria (Fig. 22)	*Saprinus* subgenus *Pilisaprinus* Kanaar, 1996
5 (4)	Lateral metaventral stria shortened, not reaching metepisternum; dorsal elytral striae weakly developed, almost obliterated by coarse and dense punctuation; carinal prosternal striae present as vague rudiments on prosternal apophysis or completely absent; lateral prosternal striae rudimentary, never joined anteriorly (for fig. see Lackner 2009: fig. 64); pronotal depressions absent	*Terametopon* subgenus *Psammoprinus* Gomy & Vienna, 1996
6 (3)	Prosternum asetose, elytral epipleuron asetose; marginal elytral stria double	*Gnathoncus* Jacquelin-Duval, 1858
7 (2)	Prosternal pre-apical foveae present (Fig. 8)
8 (9)	Prosternal pre-apical foveae small and connected by a deep sulcus (Fig. 23)	*Euspilotus* subgenus *Neosaprinus* Bickhardt, 1909
9 (8)	Prosternal pre-apical foveae well developed, deep and not connected by a deep sulcus (Fig. 24); marginal prosternal stria present anteriorly, but not connecting prosternal pre-apical foveae	*Euspilotus* subgenus *Hesperosaprinus* Bickhardt, 1909
10 (1)	At least supraorbital striae always present, often both frontal and supraorbital striae present (Fig. 4)
11 (12)	Frons with a massive frontoclypeal projection with a remnant of frontal stria (for fig. see Lackner 2009, fig. 4)	*Terametopon* subgenus *Terametopon* Vienna, 1987
12 (11)	Frons without any projection (Fig. 4)
13 (22)	Prosternal pre-apical foveae absent (for fig. see Lackner 2010, fig. 305)^2^
14 (17)	Dorsal surface almost completely impunctate; hind femora swollen (Fig. 25)
15	(16) Protibia on outer margin with two massive triangular distal teeth topped by short rounded denticle, followed by approximately ten short thin denticles diminishing in size in proximal direction (Fig. 26). Sexually dimorphic taxon with female having pointed elytral apices and first abdominal sternite projected, overlying the rest of the sternites (Fig. 25)	*Pachylopus* Erichson, 1834
16 (15)	Protibia on outer margin with approximately nine low teeth topped by large denticle gradually diminishing in size in proximal direction (Fig. 27); both sexes similar in appearance, sexual dimorphism absent	*Neopachylopus secqi* Kanaar, 1998
17 (14)	Dorsal surface usually punctate; hind femora normally not swollen (Fig. 1)
18 (19)	Lateral prosternal striae apically conspicuously hooked inwardly (Fig. 28); frontal disc with two deeply marked chevrons (Fig. 29)	*Parahypocaccus* Vienna, 1995
19 (18)	Configuration of lateral prosternal striae variable, but their apices never hooked inwardly and frontal disc without chevrons
20 (21)	Frontal stria almost absent, supraorbital stria well developed (Fig. 30), protibia with dense row of small round almost identical stout denticles on anterior and outer margins (Fig. 31); elytral disc with deep transverse rugae (Fig. 32)	*Paraphilothis* Vienna, 1994
21 (20)	Frontal stria complete or interrupted (and occasionally prolonged onto clypeus); shape of protibia variable, but never with a dense row of short identical stout denticles; elytral disc usually punctate, transverse rugae never present	*Saprinus* Erichson, 1834
22 (13)	Prosternal pre-apical foveae present (Fig. 4)
23 (28)	Prosternal pre-apical foveae connected by marginal prosternal stria (Fig. 4)
24 (25)	Pronotal hypomeron setose; body black, never metallic	*Pholioxenus*^3^ (in part) Reichardt, 1932
25 (24)	Pronotal hypomeron glabrous; body metallic or not
26 (27)	Pronotal depressions (for fig. see Lackner 2010: fig. 146; ‘pronotal fovea’) present, body in most cases metallic, dorsal punctuation not coarse or very dense	*Chalcionellus*^4^ Reichardt, 1932
27 (26)	Pronotal depressions absent, species not metallic, punctuation of dorsum very coarse and dense (Fig. 1)	*Afroprinus* gen. n.
28 (23)	Prosternal pre-apical foveae not connected by marginal prosternal stria (for fig. see Lackner 2010: fig. 148)
29 (30)	Antennal scape strongly thickened (Fig. 33)	*Dahlgrenius* Penati & Vienna, 1995
30 (29)	Antennal scape not strongly thickened (Fig. 4)
31	(32) Frontal stria widely open anteriorly, prolonged onto clypeus; dorsal elytral striae 1-3 completely erased by coarse and dense punctuation	*Saprinus (Saprinus) caeruleatus* Lewis, 1905
32 (31)	Frontal stria usually complete and often carinate; elytral striae 1-3 normally observable
33	(37) Frons coarsely and densely punctate, with numerous short rugae, occasionally with several transverse deep rugae (for fig. see Lackner 2010: fig. 420)
35 (36)	Pronotal hypomeron setose; protibia with two massive triangular distal denticles (Fig. 34); metatibia strongly thickened, outer margin with three-four rows of short denticles (Fig. 35)	*Exaesiopus* Reichardt, 1926
36 (35)	Pronotal hypomeron setose or not; protibia with four to seven low teeth topped by moderately large denticles gradually diminishing in size in proximal direction (for fig. see Lackner 2010: 461); metatibia normally not very thickened, its outer margin normally with two to three rows of denticles	*Hypocaccus* Thomson, 1867
37 (33)	Frons finely to moderately punctate (for fig. see Lackner 2010: fig. 400)
38 (39)	Pronotal hypomeron normally asetose, most of the taxa with metallic tinge	*Hypocacculus* Bickhardt, 1914
39 (38)	Pronotal hypomeron always setose, taxa almost always without metallic tinge	*Pholioxenus* Reichardt, 1932 (in part)

**Figures 22–29.**
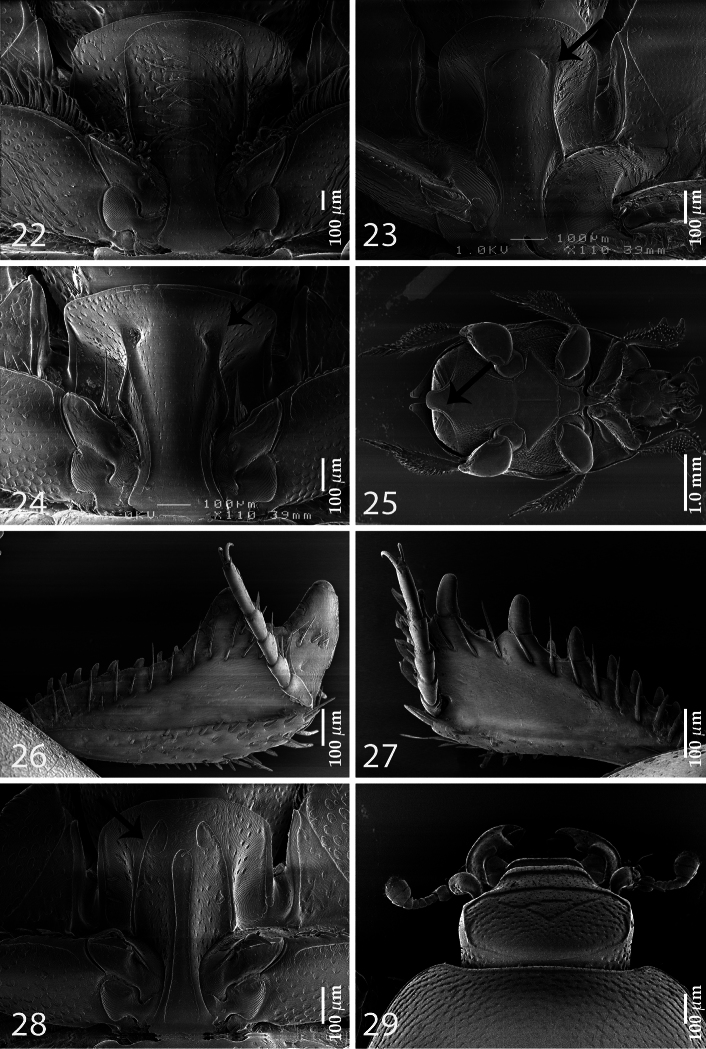
Assorted Saprininae characters. **22** *Saprinus (Pilisaprinus) verschureni* Thérond, 1959, prosternum **23** *Euspilotus (Neosaprinus) rubriculus* (Marseul, 1855), prosternum **24** *Euspilotus (Hesperosaprinus) modestus* (Erichson, 1834), prosternum **25** *Pachylopus dispar* Erichson, 1834, female, ventral view **26** *Pachylopus dispar* Erichson, 1834, protibia, dorsal view **27** *Neopachylopus secqi* Kanaar, 1998, protobia, dorsal view **28** *Parahypocaccus weyerichi* Vienna, 1995, prosternum **29** *Parahypocaccus weyerichi* Vienna, 1995, head, dorsal view.

**Figures 30–35.**
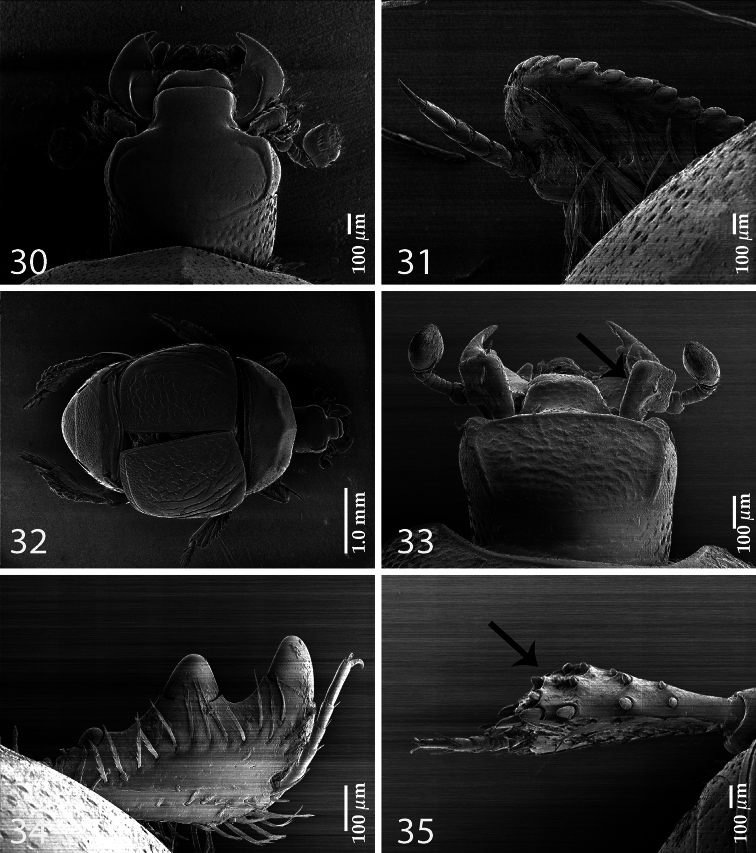
Assorted Saprininae characters. **30** *Paraphilothis mirabilis* Vienna, 1994, head, dorsal view **31** *Paraphilothis mirabilis* Vienna, 1994, protibia, dorsal view **32** *Paraphilothis mirabilis* Vienna, 1994, habitus, dorsal view **33** *Dahlgrenius aurosus* (Bickhardt, 1921), head, dorsal view **34** *Exaesiopus laevis* Thérond, 1964, protibia, dorsal view **35** *Exaesiopus laevis* Thérond, 1964, metatibia, dorsal view.

## Discussion

In the recently performed phylogenetic analysis focused on the resolving the relationships of the higher taxa of the Saprininae subfamily, this new genus has been placed within a large and unresolved clade of genera that all share a unique synapomorphy of a single, pear-shaped vesicle inside the antennal club, as well as several weaker synapomorphies, which are possibly homoplasies (Lackner, unpublished). Within that large unresolved clade, *Afroprinus* was placed in a small dichotomy, sister to a South African member of the genus *Pholioxenus*, *Pholioxenus oleolus* Thérond, 1965 that was included in the analysis to test the monophyly of the genus *Pholioxenus*. Both *Afroprinus* and *Pholioxenus oleolus* are characterized by the putatively plesiomorphic feature of prosternal pre-apical foveae connected by marginal prosternal stria. Such a feature, which might also represent a convergence, is normally present among the members of the subgenera *Hemisaprinus* and *Neosaprinus* of the genus *Euspilotus* and its absence among other members of *Pholioxenus* points to the possible polyphyly of the genus. In the analysis, *Hemisaprinus* and *Neosaprinus* came out closer to the root of the cladogram unrelated to the large clade mentioned above.

Cavernicolous habits are not common in the Histeridae, with most of the troglophilous genera belonging to subfamilies and tribes containing mostly microhisteridae: Dendrophilinae: Bacaniini (e.g. genera *Troglobacanius* Vomero, 1974, or *Sardulus* Patrizi, 1955), Abraeinae: Abraeini (genus *Spelaeabraeus* Moro, 1957), Acritini (genus *Spelaeacritus* Jeannel, 1934), see also Kovarik and Caterino (2005) for more thorough enumeration of the cavernicolous histerids. *Speleacritus anophtalmus* Jeannel, 1934 even shares some of the morphological adaptations that are typical of cavernicolous beetles: elongate body appendages, quasi-prognathous head, and fused elytra (Kovarik and Caterino 2005). In the Saprininae there are currently several genera whose species are known to have been collected inside caves: *Gnathoncus* Jacquelin du Val, 1858 (*Gnathoncus cerberus* Auzat, 1923 and *Gnathoncus cavicola* Normand, 1949), *Tomogenius* Marseul, 1862 (*Tomogenius incisus* (Erichson, 1842); *Tomogenius ripiciola* (Marseul, 1870); *Tomogenius motocola* Mazur, 1990 and *Tomogenius papuaensis* Gomy, 2007)), *Euspilotus* Lewis, 1905 (*Euspilotus (Neosaprinus) rubriculus* (Marseul 1855); *Euspilotus (Neosaprinus) scrupularis* (J.E. LeConte, 1859); *Euspilotus (Neosaprinus) burgeoisi* (Desbordes, 1920); *Euspilotus (Neosaprinus) turikensis* Kanaar, 1993 and *Euspilotus (Hesperosaprinus) modestus* Erichson, 1834 and *Euspilotus (Hesperosaprinus) sterquilinus* (J.L. LeConte, 1859) (Kovarik & Caterino 2005 and Lackner, unpublished)). None of these taxa exhibit any morphological adaptations to the cavernicolous way of life and according to Kovarik & Caterino (2005), they are attracted to bat guano accumulated inside these caves and presumably feed on fly larvae developing in it. The habitat of *Afroprinus*, which is similar to the genera mentioned above, is atypical for the members of the large and poorly resolved clade of the yet unpublished phylogeny of the Saprininae subfamily. Typically, its other members, such as the genus *Chalcionellus* are predators inhabiting open areas and are normally found on dung or carcasses in dry or semiarid steppe zones, on shoals of rivers or beaches. This group contains all of the hitherto known true psammophiles, as well as an obligate termitoxene.

Regarding the Saprininae collected in caves in the Afrotropical region apart from *Afroprinus cavicola*, a single non-native species, *Euspilotus (Neosaprinus) rubriculus* (Marseul, 1855) has also been collected inside a cave. This Neotropical species (for details on its distribution see Mazur 2011: 192) has been introduced to the French island of La Réunion in the Indian Ocean and to the British Overseas Territory of Saint Helene in the Atlantic Ocean (Desbordes 1919; Thérond 1972; Gomy 2005). Specimens collected on La Réunion have been found inside a lava tube in the faeces of the Mascarene Swiftlet (*Collocalia francica* Gmelin, 1789) (Gomy 2005). Gomy (2005) concluded that its cavernicolous habitat “*n’a rien d’exceptionel*” and hypothesized that this species could have come from Brazil with a shipment of some kind of legumes, probably soy beans or corn. The species seems to be well established on the island, since it has been collected in the same environment again (Gomy 2005). Perhaps the above-mentioned Saprininae taxa (including *Afroprinus*) that have been collected inside caves show an early stage of colonisation of the cave environment by Saprininae histerids. Saprininae are one of the most ecologically plastic histerids, occurring inside mammals’ burrows, ant nests, on decomposing vegetation, on coastal wrack, deep under shifting sands, and even on flowering plants (see Lackner 2010 for details on Saprininae biology). The discovery of this peculiar new genus inside an African cave proves that our knowledge of Afrotropical Saprininae is still incomplete.

## Supplementary Material

XML Treatment for Afroprinus

XML Treatment for Afroprinus cavicola

## Endnotes

1 The species *Xenonychus tridens* (Jacquelin du Val, 1852) also known from several sub-Saharan countries (Chad, Mauritania and Niger) as well as from the Cape Verde Archipelago has variable frontal and supraorbital striae, which are mostly present. This species possesses well-developed prosternal pre-apical foveae and setose elytral epipleuron. The other African species, *Xenonychus somaliensis* (Thérond, 1963) known only from Somalia, lacks frontal and supraorbital striae altogether, but has likewise well-developed pre-apical foveae and setose elytral epipleuron (see also Lackner 2012 for details).

2 Species *Neopachylopus secqi* Kanaar, 1998 recorded from Djibouti possesses extremely small pre-apical foveae visible only with strong magnification.

3 Several species of *Pholioxenus* do not have their pre-apical foveae connected by marginal prosternal stria. In this regard, they resemble species of the genus *Hypocacculus*. Limits of both genera are unclear and highly subjective, pending the revision of both genera.

4 Exception *Chalcionellus io* Mazur, 1993.
